# Knowledge, attitude, and practice of Chinese parents with infants (aged 0–3 years) toward immunity, gut microbiota and biotics: a comprehensive study

**DOI:** 10.3389/fimmu.2024.1396087

**Published:** 2024-07-15

**Authors:** Jiongnan Wang, Nan Liu, Yufan Chen, Jialu You, Yunqing Yang, Yi Jin, Guiju Sun, Jin Zhou

**Affiliations:** ^1^ Key Laboratory of Environmental Medicine and Engineering of Ministry of Education/Department of Nutrition and Food Hygiene, School of Public Health, Southeast University, Nanjing, China; ^2^ Chinese Nutrition Center for Education, Education and Training Center of Chinese Nutrition Society, Beijing, China; ^3^ Danone Open Science Research Center for Life-Transforming Nutrition, Shanghai, China

**Keywords:** infants, KAP, immunity, gut microbiota, biotics

## Abstract

In the wake of the COVID-19 pandemic, there has been an increasing focus towards infant immunity. The development and maintenance of the immune system are significantly influenced from birth, and is shaped by early-life infant feeding behavior. Hence, the knowledge, attitude, and practice (KAP) of parents play a crucial role in shaping the immune system of infants. A total of 2369 parents across 19 cities in China were surveyed using a self-designed online questionnaire. The KAP questionnaire assessed three domains: immunity, gut microbiota, and biotics (prebiotics, probiotics, and synbiotics). The questionnaire also included questions on infant health. An overwhelming majority of parents (97.90%) placed high value on their children’s immunity, and 40.40% of them reported an increased level of concern following the COVID-19 pandemic. Diarrhea (78.80%), colds (75.70%), nighttime crying (73.80%), regurgitation (71.70%) and food retention (66.50%) were the major health issues reported. Knowledge scores toward immunity and gut microbiota were positively correlated to attitude and practice scores, respectively. Attitude scores toward immunity, gut microbiota and biotics were positively correlated to their respective practice scores. Parental knowledge score on immunity was negatively correlated with diarrhea, colds, regurgitation and food retention. On the other hand, parental practice scores toward immunity were negatively correlated with food retention. In regards to gut microbiota, parental knowledge score was negatively correlated with diarrhea, regurgitation and food retention; parental attitude score was negatively correlated with nighttime crying whereas practice score was negatively correlated with diarrhea, regurgitation, food retention and nighttime crying. Attitude score toward biotics was negatively correlated with nighttime crying and practice scores toward biotics was negatively correlated with colds, food retention and nighttime crying. This study demonstrated that significant gaps and misunderstandings exist among parents regarding immunity, gut microbiota health, and biotics. Both public education and interventions are crucial to enhance parental knowledge and practices, thereby improving infant immunity.

## 1 Introduction

In this post-COVID-19 era, there has been a heightened focus on the immune health of infants. Infants are susceptible to external infections due to their immature autoimmune function, whereby their immune system can be influenced by bacteria, viruses, and other pathogens. Host-microbial synbiosis contributes to homeostasis and immune function regulation (1, 2). Several epidemiological studies have identified a distinct correlation between childhood gut microbiota disruptions and the development of immune and metabolic disorders in later life (3–5). The onset of autoimmune diseases and allergies were allegedly associated to altered gut microbiota development (6). Maturation of the immune system after birth is dependent on intestinal microbiome composition; its development, microbiome diversity, and intestinal function significantly influenced by the type of feeding (7). These emphasize the significance of parental KAP toward infant feeding, as they play a crucial role in influencing the development of infant immune system.

The “hygiene hypothesis” highlights that reduced exposure to external stimuli such as parasites and microbes during infancy and/or childhood can result in an overreaction of the immune system, potentially increasing the risk of allergies and autoimmune diseases and therefore not conducive to the maturation of the immune system (8). In recent years, the COVID-19 epidemic has restricted infants’ exposure to external stimuli necessary for immune training, thus diminishing the body’s ability to respond to complex external environments such as influenza. The goal of this study is to assess parental health literacy and understanding on infant immunity, principles of preventive immunology, and identify future opportunities for parental education to help infants build stronger immunity to navigate the challenges posed in this post-COVID-19 era.

## 2 Materials and methods

### 2.1 Study design

Parents with infants aged 0–3 years old across 19 Chinese cities (Shanghai, Beijing, Shenzhen, Suzhou, Chengdu, Nanjing, Hangzhou, Tianjin, Guangzhou, Chongqing, Wuhan, Xi’an, Hefei, Changzhou, Fuzhou, Changsha, Ningbo, Qingdao, and Harbin) were invited to participate in the survey. Different tiered cities (first and second), as well as coastal and inland cities were chosen to ensure a comprehensive representation in the survey. Data was collected using Credamo Inc through an online questionnaire from 12th September 2023 to 12th November 2023. The platform was chosen considering it is popular and well-established in conducting survey research in China at the time of data collection. It also supports questionnaires of similar types, participant screening procedures, as well as incentive arrangement and distribution. Exclusion criteria include: (1) questionnaire with a response time of less than 5 min, and (2) questionnaires filled by grandparents and/or parents aged ≥ 50 years old. Participation in this study was anonymous, confidential and voluntary.

### 2.2 Questionnaire design

This study used a questionnaire based on the KAP model (9, 10). The questionnaire consists of 54 questions on the demographic profile of participants (parents and infants aged 0–3 years) and parental KAP on three major areas including immunity, gut microbiota and biotics. The reliability coefficient for the final questionnaires was 0.923 (Cronbach’s α). After repeated measurements, stability coefficients of total scores on knowledge, attitude and practice were 0.723, 0.727 and 0.357, respectively (P<0.05).

### 2.3 Scoring

The KAP questionnaire included questions on the basic information of parents and infants, infant problems over the past 3 months, and questions related to immunity, gut microbiota, and biotics. There were 15 knowledge questions, 21 attitude questions and 8 practice questions. Ratio of knowledge, attitude and practice questions on immunity, gut microbiota and biotics were 5:19:1, 6:1:1, and 4:1:6, respectively. Knowledge questions were either single-choice or multiple-choice questions, and each correctly answered question was assigned a value of 1 point. Two variations of attitude questions were designed: a four-level scale (Unclear/Uncertain = 1 point, No Influence = 2 points, Some Influence = 3 points, Great Influence = 4 points) and a five-level scale ranging from “No Impact” (1 point) to “Very Big Impact” (5 point). Practice questions were five-level scale (Never use = 1 point, ≤ 1 times/week = 2 points, 2–3 times/week = 3 points, ≥ 4 times/week = 4 points, make and persist for 3 weeks or more every day = 5 points) and multiple-choice questions. Scoring for multiple-choice questions were calculated as follow: 1/Total number of options selected.

### 2.4 Data analysis

Statistical analyses were performed using IBM SPSS 25.0 software. Statistical analysis including descriptive statistics, correlation analysis and regression analysis were performed on the demographic characteristics of participants; Spearman’s correlation coefficient was used for correlation analysis. Statistical significance was defined as p <0.05.

### 2.5 Ethical approval

The study was approved by the Ethics Committee of Capital Medical University, the ethical approval number is Z2023SY085.

## 3 Results

### 3.1 Basic information of parents and infants

Demographic profile of 2369 parents who participated in this study were presented in **Table 1**. Out of the total participants, 2084 resided in urban areas and 600 in rural areas. A higher number of women (n=1359) participated in this study compared to men (n=1010). Number of parents aged 19–29, 30–39, 40–49 years were 874, 1333 and 162, respectively. There were 75 single-family households, 1694 nuclear families, and 600 extended families in the study. In terms of education level, 5 had primary school education or lower, 45 had junior high school education, 158 had high school or technical school education, 1711 had either finished junior college or obtained an undergraduate degree, and 1711 had at least a master’s degree. Monthly income of parents is as follows: 45 had a monthly income of less than 2500 CNY, 421 had a monthly income from 2500 to 5000 CNY, 1126 had a monthly income between 5000 and 10000 CNY, and 777 had a monthly income exceeding 10000 CNY.

**Table 1:** Baseline information of surveyed parents.

Demographic characteristics		Total	Percentage (%)
Relationship	Father	1010	42.63
	Mother	1359	57.37
Age	19–29	874	36.89
	30–39	1333	56.27
	40–49	162	6.84
Place of residence	Urban area	2084	87.97
	Rural areas	285	12.03
Family structure	Single-family	75	3.17
	Nuclear family	1694	71.51
	Extended family (three or more generations in one household)	600	25.33
Education level	Primary school or lower	5	0.21
	Junior high school	45	1.90
	High school or technical school	158	6.67
	Junior college or bachelor degree	1711	72.22
	Master’s degree or above	450	19.00
Profession	Workers	110	4.64
	Farmers	11	0.50
	Clerks	992	41.87
	Science, education, cultural and health sector personnel	193	8.15
	Civil servant	98	4.13
	Business manager	616	26.00
	Sole proprietorship and cooperatives	104	4.39
	Freelance worker	145	6.12
	Others	100	4.22
Monthly income	<2500 CNY	45	1.90
	2500–5000 CNY	421	17.78
	5000–10000 CNY	1126	47.53
	>10000 CNY	777	32.80

Baseline characteristics of infants (0–3 years) were shown in **Table 2**. Male infants make up 52.40%, slightly outnumbering female infants at 47.60%. The proportions of infants aged 0–6 months, 7–12 months, and 1–3 years were 7.80%, 21.00%, and 71.30%, respectively.

**Table 2:** Basic information of infants aged 0–3 years old.

Age group	Male (%)	Female (%)
0–6 months	100 (54.30)	84 (45.70)
7–12 months	257 (51.7)	240 (48.3)
1–3 years	884 (52.40)	804 (47.60)
Total (%)	1241 (52.40)	1128 (47.60)

Data presented as n (%).

### 3.2 Parental attention towards infant immunity pre- and post- COVID 19

**Table 3** showed the general parental attention towards infant immunity pre- and post- COVID 19. Majority of parents (57.50%) responded that they have always attached great importance to infant immunity regardless of the COVID-19 outbreak. 40.40% of parents showed increased attention whereas 0.50% of parents showed reduced attention to infant immunity post-COVID 19. Conversely, 1.50% of parents indicated that they never put much thought into this.

**Table 3:** Survey on parental attention towards infant immunity pre- and post-COVID-19.

Age group	Increased attention (%)	Always attached great importance (%)	Decreased attention (%)	Never paid much attention (%)
0–6 months	80 (43.50)	101 (54.90)	2 (1.10)	1 (0.50)
7–12 months	198 (39.80)	285 (57.30)	5 (1.00)	9 (1.80)
1–3 years	680 (40.30)	976 (57.80)	6 (0.40)	26 (1.50)
Total	958 (40.40)	1363 (57.50)	13 (0.50)	36 (1.50)

Data presented as n (%).

### 3.3 Parent-reported infant health problems

Major parent-reported infant health problems across infants of all age group were diarrhea (n = 1867), colds (n = 1794), nighttime crying (n = 1748), regurgitation (n = 1699) and food retention (n =1576). Diarrhea was identified as the primary health problem among infants aged 0–6 months and 1–3 years, while regurgitation was the primary health issue in infants of 7–12 months (**Table 4**).

**Table 4:** Parent-reported infants health problems.

Health problems		Age group			Total
		0–6 months	7–12 months	1–3 years	
Skin	Eczema	81(44.00)	257(51.70)	797(47.20)	1135(47.90)
	Urticaria	21(11.40)	74(14.90)	205(12.10)	300(12.70)
	Other	117(63.60)	316(63.60)	1010(59.80)	1443(60.90)
Respiratory tract	Cold	119(64.70)	363(73.00)	1312(77.70)	1794(75.70)
	Rhinitis	28(15.20)	91(18.30)	317(18.80)	436(18.40)
	Pharyngitis	27(14.70)	95(19.10)	288(17.10)	410(17.30)
	Bronchitis	19(10.30)	116(23.30)	381(22.60)	516(21.80)
	Pneumonia	19(10.30)	73(14.70)	244(14.50)	336(14.20)
Digestive tract	Vomiting	99(53.80)	299(60.20)	886(52.50)	1284(54.20)
	Diarrhea (≥3 times a day)	140(76.10)	410(82.50)	1317(78.00)	1867(78.80)
	Gastritis	14(7.60)	56(11.30)	140(8.30)	210(8.90)
	Enteritis	26(14.10)	79(15.90)	250(14.80)	355(15.00)
	Regurgitation	166(90.20)	425(85.50)	1108(65.60)	1699(71.70)
	Milk vomiting	138(75.00)	346(69.60)	886(52.50)	1370(57.80)
	Food retention	113(61.40)	332(66.80)	1131(67.00)	1576(66.50)
	Flatulence	112(60.90)	294(59.20)	945(56.00)	1351(57.00)
	Hard stool	87(47.30)	302(60.80)	978(57.90)	1367(57.70)
	Colic	43(23.40)	112(22.50)	212(12.60)	367(15.50)
Other	Fever	93(50.50)	294(59.20)	1012(60.00)	1399(59.10)
	Nighttime crying	155(84.20)	414(83.30)	1179(69.80)	1748(73.80)
	Allergy	31(16.80)	116(23.30)	339(20.10)	486(20.50)

Data presented as n (%).

### 3.4 Knowledge, attitude and behavior of parents

#### 3.4.1 Knowledge survey

Majority of parents (89.50%) considered that infancy is a crucial period for establishing a robust immunity in infants; 72.20% believed that nutrition during the early stages after birth is vital for the long-term development of infants’ immunity. However, 12.50% of parents believed that immunity is inherited and is influenced to a limited extent by external factors after birth. 24.80% also believed that nutritional intervention is only effective in improving the baby’s immunity in the short term. In terms of understanding the gut microbiota, only 17.10% of individuals are aware that optimizing gut microbiota can have lasting effects up to six months to a year. In regards to biotics, 95.70% of parents have a basic understanding about the term “probiotics”, but lack knowledge when it comes to identifying high-quality probiotic products as well as demonstrated a limited understanding of synbiotics [a mixture comprising of live microorganisms and substrate(s) which can be selectively utilized by host microorganisms that confers a health benefit on the host (11)] (**Table 5**).

**Table 5:** Parental knowledge toward immunity, gut microbiome and biotics.

	Parents with infants aged			
	0–6 months	7–12 months	1–3 years	Total
Immunity knowledge problem				
**1. What concepts do you believe accurately describe your child’s immunity?**				
Immunity is inherited and is influenced to a limited extent by external factors after birth.	24 (13.00)	72 (14.50)	201 (11.90)	297 (12.50)
Infancy is a critical period for building a robust immunity.	161 (87.50)	427 (85.90)	1533 (90.80)	2121 (89.50)
Immunity can be regulated by gut microbiota.	103 (56.00)	326 (65.60)	1042 (61.70)	1471 (62.10)
Nutritional intervention is only effective in improving the baby’s immunity in the short term.	39 (21.20)	129 (26.00)	420 (24.90)	588 (24.80)
Nutrition provided in the early stages after birth is crucial for the long-term immune development of infants.	130 (70.70)	332 (66.80)	1249 (74.00)	1711 (72.20)
Gut microbiota				
**1. What is gut microbiota?**				
They are probiotics.	17 (9.20)	43 (8.70)	142 (8.40)	202 (8.50)
It is helpful to maintain normal intestinal function.	159 (86.40)	441 (88.70)	1505 (89.20)	2105 (88.90)
It has little effect on health.	4 (2.20)	3 (0.60)	14 (0.80)	21 (0.90)
I don’t know.	4 (2.20)	10 (2.00)	27 (1.60)	41 (1.70)
**2. How long do you think the effects of gut microbiota optimization can last?**				
Less than one month.	24 (13.00)	28 (5.60)	134 (7.90)	186 (7.90)
1–3 months.	88 (47.80)	195 (39.20)	665 (39.40)	948 (40.00)
4–6 months.	36 (19.60)	161 (32.40)	468 (27.70)	665 (28.10)
6 months to 1 year.	24 (13.00)	84 (16.90)	297 (17.60)	405 (17.10)
≥ 1 year.	12 (6.50)	29 (5.80)	124 (7.30)	165 (7.00)
Biotics				
**1. What are probiotics?**				
They are a single type of bacteria.	6 (3.30)	7 (1.40)	22 (1.30)	35 (1.50)
They are only found in healthy people.	4 (2.20)	8 (1.60)	13 (0.80)	25 (1.10)
It is a type of beneficial bacteria found in the gut that helps in regulating gut microbiota and enhancing digestive functions within the intestinal tract.	170 (92.40)	472 (95.00)	1642 (96.20)	2266 (95.70)
I don’t know.	4 (2.20)	10 (2.00)	29 (1.70)	43 (1.80)
**2. What, in your opinion, constitutes a good probiotic product for an infant?**				
The higher the number of live probiotics, the better.	71 (38.60)	195 (39.20)	721 (42.70)	987 (41.70)
The greater the variety, the better.	38 (20.70)	127 (25.60)	424 (25.10)	589 (24.90)
They have a positive effect.	122 (66.30)	380 (76.50)	1276 (75.60)	1778 (75.10)
The probiotic strain originates from breast milk.	69 (37.50)	202 (40.60)	623 (36.90)	894 (37.70)
Verified by clinical studies	100 (54.30)	276 (5.50)	941 (55.70)	1317 (55.60)
**3. What are prebiotics?**				
A type of probiotics.	82 (44.60)	246 (49.50)	811 (48.00)	1139 (48.10)
They can be utilized by all the beneficial bacteria in the gut.	121 (65.80)	358 (72.00)	1197 (70.90)	1676 (70.70)
They are not absorbed by the human body, but provide health benefits.	91 (49.50)	238 (47.90)	803 (47.60)	1132 (47.80)
I don’t know.	22 (12.00)	38 (7.60)	101 (6.00)	161 (6.80)
**4. Please select the statement regarding the concept and function of synbiotics that you believe is correct.**				
The combination of probiotics and prebiotics is considered a synbiotic element.	25 (13.60)	64 (12.90)	271 (16.10)	360 (15.20)
Synbiotic combinations require specific probiotics and prebiotics.	63 (34.20)	185 (37.20)	557 (33.00)	805 (34.00)
Probiotics and prebiotics have synergistic effects.	71 (38.60)	211 (42.50)	729 (43.20)	1011 (42.70)
I don’t know.	25 (13.60)	37 (7.40)	131 (7.80)	193 (8.10)

Data presented as n (%).

#### 3.4.2 Parental perspectives on infants’ immunity

Majority of parents (58.10%) believed that their infants’ immunity is good, whereas 15.9% consider their infants’ immune health to be very good. 26% of parents consider their infants’ immunity levels to be average or below average.

### 3.5 Correlation between parental knowledge, attitude and practice toward immunity, gut microbiota and biotics

Correlation tests were conducted on parental KAP scores (**Table 6**). Knowledge scores toward immunity and gut microbiota were positively correlated to attitude (r=0.407, P<0.01; r=0.206, P<0.01) and practice (r=0.435, P<0.01; r=0.206, P<0.01) scores, respectively.

**Table 6:** Correlation test between knowledge and attitude scores.

	Knowledge score (immunity/gut microbiota/biotics)			
Factor	0–6 months	7–12 months	1–3 years	Total score of knowledge
Attitude score on immunity	0.471**	0.391**	0.405**	0.407**
Attitude score on gut microbiota	0.276**	0.219**	0.193**	0.206**
Attitude score on biotics	-0.045	-0.038	0.003	-0.009
Practice score on immunity	0.418**	0.470**	0.428**	0.435**
Practice score on gut microbiota	0.499**	0.461**	0.434**	0.446**
Practice score on biotics	0.042	-0.002	-0.027	-0.014
N	184	497	1688	2369

**P<0.01. P-value<0.05 significant.

Correlation tests were also performed between parental attitude and practice scores. Attitude scores were found to be positively correlated to their respective practice scores (Immunity: 0.502, P <0.01; gut microbiota:.0.200, P<0.01; biotics: 0.153, P<0.01) (**Table 7**).

**Table 7:** Correlation test between attitude and practice scores.

	Practice score (immunity/gut microbiota/biotics)			
Factor	0–6 months	7–12 months	1–3 years	Total score
Attitude score on immunity	0.519**	0.493**	0.503**	0.502**
Attitude score on gut microbiota	0.160*	0.174**	0.208**	0.200**
Attitude score on biotics	0.089	0.187**	0.151**	0.153**
N	184	497	1688	2369

*P<0.05, **P<0.01. P-value<0.05 significant.

### 3.6 Correlation between parental KAP and infant health problems

Parental practice score on gut microbiome was negatively correlated to incidence of diarrhea and food retention in infants across all age groups. On the other hand, parental practice score on biotics was negatively correlated with colic. In infants aged 0–6 months, parental knowledge score on immunity was negatively correlated to the incidence of flatulence and colic whereas attitude score was negatively correlated to colic in infants aged 0–6 months. Parental practice score on gut microbiota was negatively correlated to vomiting, food retention and hard stool; practice score on biotics was negatively correlated to pneumonia, hard stool, nighttime crying, colic and allergy, respectively. Parental KAP scores were more significantly correlated to health problems in infants aged 7–12 months. Skin redness and itching were negatively correlated with parental knowledge and practice scores on immunity. Diarrhea and nighttime crying were negatively correlated with parental practice score on immunity. In terms of gut microbiota, KAP scores were negatively correlated to vomiting, milk vomiting and vomiting and enteritis, respectively. Rhinitis, pharyngitis, bronchitis, pneumonia, gastritis, enteritis, hard stool, and colic were negatively correlated with parental practice score on biotics. Parental practice score on biotics was negatively correlated to colic whereas parental score on gut microbiota was negatively correlated with diarrhea and vomiting in infants of 1–3 years old (**Supplementary Tables 1**-**4**).

## 4 Discussion

This study indicated that most parents (97.9%) placed high value on their infants’ immunity, and 40.40% of them reported a heightened concern following the COVID-19 pandemic. More than two-thirds of parents also acknowledged that infancy and early childhood are a critical period for the development of the immune system. Research has shown that a positive interaction between gut microbiota and the immune system plays a crucial role in the formation and improvement of infant immunity (12); however, nearly 40% of parents involved in this study were not aware of this.

The assessment revealed gaps in parental knowledge, especially on the definition and selection of biotics. Almost none of the parents surveyed are aware that good probiotics require a high level of safety, functionality, and technical feasibility in accordance to the recommendations of World Health Organization (WHO), Food and Agriculture Organization (FAO), and European Food Safety Authority (EFSA) (13–15). An intervention study on infants born via caesarean suggested that synbiotics could support gut microbiota diversity and the colonization of beneficial bacteria in the gut, as well as reduce colonization of harmful bacteria; thereby promoting a healthy intestinal physiology (16, 17). However, less than half of the parents were aware that the term ‘synbiotics’ is reserved for use when probiotics and prebiotics jointly produce synergistic or complementary health effects. The disparity between research evidence and parental knowledge highlights the necessity for effective health and nutrition education. This can be achieved through research-proven techniques, such as Information, Motivation, and Behavior Skills models, a combination of lectures with brainstorming and demonstrations, as well as Brief Strategic Family Therapy (BSTF) and e-health education delivered through a mobile application (18–20).

In this study, correlation analysis revealed that higher parental knowledge on immunity was associated with an increased appreciation of its importance, hence motivating parents to make greater efforts to improve their infants’ immunity. For example, parental practice to regulate gut microbiota was positively correlated to their knowledge on gut microbiota. A study on oral health among children in Morocco yielded similar findings, suggesting that mothers with greater knowledge and more positive attitudes towards oral health are more likely to adopt healthy oral care practices on their children (21). Similarly, another study demonstrated that the ability to change one’ s eating habit may be significantly influenced by knowledge (22). However, no correlation was identified between knowledge and practice scores on biotics. This finding was consistent with a prior study that revealed mothers continued to use probiotics despite having insufficient knowledge about them (23).

In addition to examining the correlation between KAP, this study also delved into the relationship between frequently reported infant health problems and KAP toward immunity, gut microbiota, and biotics. The most frequently reported infant immune and gastrointestinal problems in this study were largely consistent with findings from previous studies (24). Higher levels of gut microbiota-regulating practices, particularly the use of biotics, were associated with a lower self-reported incidence of gut and immune issues, such as diarrhea and colic in infants across all age groups in this study.

This finding aligns with similar studies that have identified a significant association between parental health literacy and behavior and the health outcomes of children with chronic diseases (18, 25). Relevant studies have also indicated that gut microbiota modulates immune and inflammatory system responses, and is proven to be very effective in preventing and treating diseases such as constipation, intestinal infections, asthma, and allergies (17, 26, 27). Compared to prebiotics and probiotics alone, synbiotics may be a more effective approach to regulating the gut, promoting the growth and diversity of probiotics, and supporting the immune system development of infants (16, 17, 28, 29). A cross-sectional study in Palestine found that parents have a certain degree of misunderstanding and improper use of antibiotics; whereby abuse and early discontinuation of antibiotics may have a negative impact on their children’s health (16, 30). Hence, it can be observed that parents with a better understanding of health tend to adopt a more positive attitude towards this subject, leading to increased concern for their infants’ health problems. Our findings, along with the above studies, suggested that it is necessary to improve parental knowledge regarding the proper usage of biotics, which could be a potential approach to bolster infant immunity.

Age-associated differences were observed, more significant correlations were found between infant health problems and parental KAP in infants aged 7–12 months compared to infants aged 0–6 months and 1–3 years, respectively. The limited number of correlations found in the 0–6 months age group may be attributed to the small sample size. Future studies with larger sample sizes are needed to determine whether stronger correlations exist. While the sample size is comparable to the 7–12 month age group, infants of 1–3 years old are exposed to a more complex living environment with additional factors that may obscure the analysis, leading to fewer discernible associations between KAP and health outcomes (31–33).

This study primarily reflects findings from urban parents, potentially overlooking knowledge gaps that may exist between urban and rural populations. Future research could broaden sampling framework to include a more comprehensive representation of these demographics. Additionally, it is important to note that causal relationships cannot be concluded from the observed results, given the cross-sectional design of this study. Thus, it is recommended for future studies to include prospective or interventional methodologies to elucidate the causal dynamics between KAP and health outcomes. Also, the utilization of self-reported questionnaires in this study may introduce a bias towards socially desirable responses, highlighting the need for careful interpretation of the findings. Future research should consider alternative data collection strategies to mitigate potential biases.

## 5 Conclusions

This study demonstrated increased attention to infants’ immunity in the aftermath of the COVID-19 pandemic. However, large gaps and misunderstandings toward immunity, gut microbiota health and biotics were found in parents. Positive correlations were observed between parental KAP regarding immunity and gut microbiota. An inverse correlation was observed between parental KAP and the incidence of health problems in infants. Both public education and additional interventions are crucial to enhance parental knowledge and practice, thereby improving infant immunity.

## Data availability statement

The original contributions presented in the study are included in the article/**Supplementary Material**. Further inquiries can be directed to the corresponding authors.

## Ethics statement

The studies involving humans were approved by Ethics Committee of Capital Medical University, Approval number: Z2023SY085. The studies were conducted in accordance with the local legislation and institutional requirements. Written informed consent for participation was not required from the participants or the participants’ legal guardians/next of kin because this study is an online questionnaire survey, which can only be completed with consent.

## Author contributions

JW: Data curation, Formal analysis, Writing – original draft. NL: Investigation, Data curation, Writing – review & editing. YC: Methodology, Writing – review & editing. JY: Methodology, Writing – review & editing. YY: Writing – review & editing. YJ: Methodology, Writing – review & editing. GS: Conceptualization, Supervision, Methodology, Writing – review & editing. JZ: Conceptualization, Supervision, Methodology, Formal analysis, Writing – review & editing.
