# Supplementary material for: Knowledge, attitude, and practice of Chinese parents with infants (aged 0–3 years) toward immunity, gut microbiota and biotics: a comprehensive study
Source: Front Immunol. 2024 Jul 15;15:1396087. doi: 10.3389/fimmu.2024.1396087 (PMC11284022; doi:10.3389/fimmu.2024.1396087)
Supplement: Supplementary file 1 [file DataSheet_1.docx]

Supplementary Material

**Table Supplementary 1** Correlation between parental KAP scores and health problems of infants across all age groups (0-3 years).

| **Scores** | | **Immunity** | | | **Gut microbiota** | | | **Biotics** | | |
| --- | --- | --- | --- | --- | --- | --- | --- | --- | --- | --- |
|  |  | **Knowledge** | **Attitude** | **Practice** | **Knowledge** | **Attitude** | **Practice** | **Knowledge** | **Attitude** | **Practice** |
| Skin | Eczema | -0.066** | -0.004 | 0.005 | -0.051* | -0.053** | -0.061** | -0.003 | -0.054* | -0.076** |
|  | Urticaria | 0.010 | 0.039 | 0.051* | 0.035 | -0.045* | -0.053** | 0.019 | -0.042 | -0.140** |
|  | Other | -0.144** | -0.016 | -0.045* | -0.080** | -0.024 | -0.083 | -0.050* | 0.032 | -0.049* |
| Respiratory tract | Cold | -0.085** | 0.001 | -0.033 | -0.016 | 0.001 | -0.038 | 0.018 | 0.027 | -0.053** |
|  | Rhinitis | 0.027 | 0.019 | 0.026 | 0.022 | 0.013 | 0.011 | 0.032 | 0.002 | -0.093** |
|  | Pharyngitis | -0.005 | -0.020 | 0.030 | 0.015 | -0.031 | -0.035 | 0.044* | -0.001 | -0.122** |
|  | Bronchitis | -0.027 | -0.003 | 0.029 | -0.007 | 0.001 | -0.057** | 0.041* | -0.027 | -0.104** |
|  | Pneumonia | 0.001 | 0.031 | 0.042* | 0.035 | -0.001 | -0.008 | 0.025 | -0.001 | -0.108** |
| Digestive tract | Vomiting | -0.085** | 0.012 | -0.028 | -0.069** | -0.037 | -0.140** | -0.006 | 0.006 | -0.090** |
|  | Diarrhea (≥3  times a day) | -0.079** | 0.031 | -0.036 | -0.072** | 0.007 | -0.107** | -0.024 | -0.015 | -0.025 |
|  | Gastritis (%) | 0.053* | 0.051* | 0.049* | 0.032 | -0.002 | -0.016 | 0.017 | -0.032 | -0.097** |
|  | Enteritis | -0.043* | 0.000 | 0.012 | -0.046* | -0.053* | -0.097** | 0.005 | -0.063** | -0.100** |
|  | Regurtitation | -0.074** | 0.017 | -0.010 | -0.061** | -0.016 | -0.052* | -0.028 | 0.004 | -0.007 |
|  | Milk vomiting | -0.044* | 0.012 | -0.003 | -0.034 | -0.041* | -0.081** | -0.011 | -0.021 | -0.079** |
|  | Food retention | -0.079** | -0.014 | -0.042* | -0.058** | 0.001 | -0.103** | 0.008 | 0.000 | -0.060** |
|  | Flatulence | -0.066** | 0.007 | -0.023 | -0.062** | 0.012 | -0.064** | -0.007 | 0.038 | -0.041* |
|  | Hard stool | -0.062** | -0.009 | -0.034 | -0.041* | 0.019 | -0.085** | 0.011 | 0.032 | -0.060** |
|  | Colic | -0.049* | -0.025 | 0.002 | -0.038 | -0.016 | -0.074** | 0.004 | -0.018 | -0.072** |
| Other | Fever | -0.044* | 0.016 | -0.058** | -0.057* | 0.078** | -0.025 | -0.023 | - | -0.025 |
|  | Nighttime crying | -0.007 | -0.034 | 0.016 | -0.014 | -0.047* | -0.059** | 0.013 | -0.071** | -0.142** |
|  | Allergy | -0.019 | 0.005 | 0.001 | -0.023 | -0.046* | -0.062** | -0.016 | -0.066** | -0.083** |

*P＜0.05, **P＜0.01. P-value＜0.05 significant.

**Table Supplementary 2** Correlation between parental KAP scores and health problems of infants aged 0-6 months.

| **Scores** | | **Immunity** | | | **Gut microbiota** | | | **Biotics** | | |
| --- | --- | --- | --- | --- | --- | --- | --- | --- | --- | --- |
|  |  | **Knowledge** | **Attitude** | **Practice** | **Knowledge** | **Attitude** | **Practice** | **Knowledge** | **Attitude** | **Practice** |
| Skin | Eczema | -0.061 | 0.059 | 0.077 | -0.032 | 0.052 | -0.018 | 0.007 | -0.154 | -0.027 |
|  | Urticaria | -0.109 | -0.024 | -0.006 | -0.004 | -0.004 | -0.132 | 0.072 | 0.014 | -0.102 |
|  | Other | -0.092 | -0.061 | 0.009 | -0.090 | 0.021 | 0.024 | -0.168* | -0.046 | 0.009 |
| Respiratory tract | Cold | -0.137 | -0.002 | 0.006 | -0.018 | 0.033 | -0.090 | -0.041 | 0.074 | -0.131 |
|  | Rhinitis | 0.046 | 0.065 | 0.068 | 0.077 | 0.113 | -0.015 | 0.089 | 0.062 | -0.067 |
|  | Pharyngitis | 0.047 | 0.026 | 0.070 | 0.079 | 0.180* | 0.004 | 0.049 | -0.011 | 0.016 |
|  | Bronchitis | -0.091 | 0.044 | 0.066 | 0.072 | 0.101 | -0.029 | 0.121 | 0.068 | 0.038 |
|  | Pneumonia | -0.091 | -0.138 | -0.023 | -0.108 | -0.012 | -0.118 | 0.023 | 0.095 | -0.185* |
| Digestive tract | Vomiting | -0.062 | 0.089 | -0.072 | -0.123 | 0.020 | -0.145* | -0.042 | 0.065 | -0.096 |
|  | Diarrhea (≥3  times a day) | -0.082 | 0.037 | -0.020 | -0.062 | 0.073 | -0.094 | -0.097 | -0.080 | -0.041 |
|  | Gastritis (%) | -0.010 | -0.029 | -0.016 | -0.004 | 0.056 | -0.020 | 0.057 | -0.121 | -0.104 |
|  | Enteritis | -0.072 | -0.093 | -0.010 | -0.026 | 0.027 | -0.069 | -0.077 | -0.103 | -0.112 |
|  | Regurgitation | -0.071 | -0.091 | -0.054 | -0.048 | -0.050 | 0.064 | 0.037 | -0.086 | 0.023 |
|  | Milk vomiting | -0.055 | -0.026 | -0.023 | -0.042 | -0.039 | -0.102 | -0.074 | -0.019 | -0.096 |
|  | Food retention | -0.130 | -0.009 | 0.016 | -0.008 | 0.024 | -0.155* | -0.051 | -0.020 | -0.132 |
|  | Flatulence | -0.215** | -0.030 | -0.039 | -0.049 | -0.001 | 0.001 | 0.021 | 0.114 | 0.000 |
|  | Hard stool | -0.039 | -0.092 | -0.036 | -0.115 | -0.017 | -0.147* | 0.001 | -0.107 | -0.164* |
|  | Colic | -0.063 | -0.049 | 0.052 | 0.002 | 0.000 | -0.086 | 0.002 | -0.014 | -0.127 |
| Other | Fever | -0.131 | -0.110 | -0.038 | -0.144 | -0.006 | -0.091 | -0.060 | -0.021 | -0.136 |
|  | Nighttime crying | -0.183* | -0.223** | -0.093 | -0.114 | -0.057 | -0.121 | 0.068 | -0.027 | -0.184* |
|  | Allergy | -0.083 | -0.021 | -0.051 | -0.019 | -0.044 | -0.116 | 0.030 | -0.151 | -0.167* |

*P＜0.05, **P＜0.01. P-value＜0.05 significant.

**Table Supplementary 3** Correlation between parental KAP scores and health problems of infants aged 7-12 months.

| **Scores** | | **Immunity** | | | **Gut microbiota** | | | **Biotics** | | |
| --- | --- | --- | --- | --- | --- | --- | --- | --- | --- | --- |
|  |  | **Knowledge** | **Attitude** | **Practice** | **Knowledge** | **Attitude** | **Practice** | **Knowledge** | **Attitude** | **Practice** |
| Skin | Eczema | -0.094* | -0.032 | -0.026 | -0.110* | -0.124** | -0.085 | 0.022 | -0.046 | -0.081 |
|  | Urticaria | 0.046 | 0.063 | 0.056 | 0.064 | -0.038 | 0.016 | 0.042 | -0.095* | -0.142** |
|  | Other | -0.194** | -0.099* | -0.159** | -0.111* | -0.111* | -0.062 | 0.036 | -0.005 | -0.025 |
| Respiratory tract | Cold | -0.163** | -0.060 | -0.076 | -0.042 | -0.034 | -0.008 | 0.055 | 0.047 | -0.123** |
|  | Rhinitis | 0.046 | 0.037 | 0.098* | 0.081 | 0.007 | 0.025 | 0.038 | -0.065 | -0.177** |
|  | Pharyngitis | 0.004 | -0.056 | 0.078 | 0.082 | -0.052 | -0.034 | 0.015 | -0.007 | -0.235** |
|  | Bronchitis | -0.017 | -0.029 | 0.098* | 0.036 | -0.086 | -0.052 | 0.090* | -0.074 | -0.179** |
|  | Pneumonia | 0.020 | 0.054 | 0.079 | 0.074 | -0.046 | 0.010 | 0.058 | -0.045 | -0.150** |
| Digestive tract | Vomiting | -0.115* | -0.110* | -0.097* | -0.154** | -0.089* | -0.181** | 0.015 | -0.023 | -0.091* |
|  | Diarrhea (≥3  times a day) | -0.135** | -0.053 | -0.167** | -0.101* | -0.049 | -0.104* | 0.054 | -0.013 | -0.035 |
|  | Gastritis (%) | 0.047 | -0.006 | 0.069 | 0.048 | -0.008 | -0.044 | 0.002 | -0.046 | -0.183** |
|  | Enteritis | -0.047 | -0.047 | -0.057 | -0.138** | -0.084 | -0.154** | -0.046 | -0.139** | -0.181** |
|  | Regurgitation | -0.087 | -0.034 | -0.141** | -0.073 | -0.090* | -0.109* | 0.012 | 0.024 | 0.013 |
|  | Milk vomiting | -0.100* | -0.064 | -0.104* | -0.090* | -0.157** | -0.100* | 0.076 | -0.010 | -0.121** |
|  | Food retention | -0.087 | -0.087 | -0.137** | -0.100* | -0.065 | -0.098* | 0.052 | 0.029 | -0.013 |
|  | Flatulence | -0.075 | -0.032 | -0.085 | -0.052 | -0.093* | -0.034 | 0.044 | 0.013 | -0.018 |
|  | Hard stool | -0.100* | -0.083 | -0.113* | -0.049 | -0.068 | -0.176** | 0.075 | -0.055 | -0.156** |
|  | Colic | -0.077 | -0.067 | -0.022 | -0.086 | -0.043 | -0.113* | 0.019 | 0.022 | -0.127** |
| Other | Fever | -0.081 | -0.021 | -0.153** | -0.044 | -0.044 | -0.015 | 0.032 | 0.080 | -0.013 |
|  | Nighttime crying | 0.065 | 0.022 | 0.046 | 0.015 | -0.073 | -0.024 | -0.038 | -0.130** | -0.236** |
|  | Allergy | -0.049 | -0.038 | -0.054 | -0.055 | -0.108* | -0.069 | -0.046 | -0.109* | -0.131** |

*P＜0.05, **P＜0.01. P-value＜0.05 significant.

**Table Supplementary 4** Correlation between parental KAP scores and health problems of infants aged 1-3 years.

| **Scores** | | **Immunity** | | | **Gut microbiota** | | | **Biotics** | | |
| --- | --- | --- | --- | --- | --- | --- | --- | --- | --- | --- |
|  |  | **Knowledge** | **Attitude** | **Practice** | **Knowledge** | **Attitude** | **Practice** | **Knowledge** | **Attitude** | **Practice** |
| Skin | Eczema | -0.056* | 0.00 | 0.004 | -0.033 | -0.047 | -0.056* | -0.011 | -0.045 | -0.082** |
|  | Urticaria | 0.012 | 0.038 | 0.055* | 0.031 | -0.054* | -0.065** | 0.006 | -0.031 | -0.142** |
|  | Other | -0.136** | 0.015 | -0.019 | -0.069** | -0.007 | -0.107** | -0.064** | 0.051* | -0.068** |
| Respiratory tract | Cold | -0.054* | 0.022 | -0.024 | -0.007 | 0.010 | -0.041 | 0.016 | 0.015 | -0.013 |
|  | Rhinitis | 0.020 | 0.010 | 0.001 | -0.001 | 0.007 | 0.010 | 0.025 | 0.016 | -0.071** |
|  | Pharyngitis | -0.013 | -0.014 | 0.011 | -0.012 | -0.047 | -0.039 | 0.054* | 0.003 | -0.101** |
|  | Bronchitis | -0.025 | 0.000 | 0.005 | -0.024 | 0.022 | -0.053* | 0.023 | -0.018 | -.086** |
|  | Pneumonia | 0.004 | 0.041 | 0.038 | 0.039 | 0.015 | 0.002 | 0.016 | 0.006 | -0.083** |
| Digestive tract | Vomiting | -0.077** | 0.041 | -0.006 | -0.036 | -0.035 | -0.129** | -0.009 | 0.010 | -0.093** |
|  | Diarrhea (≥3  times a day) | -0.059* | 0.057* | -0.001 | -0.066** | 0.007 | -0.114** | -0.039 | -0.009 | -0.027 |
|  | Gastritis (%) | 0.063** | 0.078** | 0.048 | 0.031 | -0.008 | -0.003 | 0.019 | -0.017 | -0.068** |
|  | Enteritis | -0.037 | 0.024 | 0.034 | -0.021 | -0.054* | -0.083** | 0.030 | -0.035 | -0.073** |
|  | Regurgitation | -0.070** | 0.040 | 0.023 | -0.066** | -0.019 | -0.066** | -0.046 | 0.010 | -0.044 |
|  | Milk vomiting | -0.025 | 0.041 | 0.027 | -0.019 | -0.026 | -0.085** | -0.030 | -0.030 | -0.086** |
|  | Food retention | -0.069** | 0.007 | -0.021 | -0.052* | 0.018 | -0.099** | 0.002 | -0.007 | -0.064** |
|  | Flatulence | -0.044 | 0.021 | -0.002 | -0.066** | 0.039 | -0.088** | -0.026 | 0.036 | -0.060* |
|  | Hard stool | -0.053* | 0.021 | -0.012 | -0.029 | 0.049* | -0.045 | -0.005 | 0.074** | -0.013 |
|  | Colic | -0.038 | -0.008 | 0.002 | -0.026 | -0.010 | -0.059* | 0.001 | -0.029 | -0.044 |
| Other | Fever | -0.021 | 0.039 | -0.035 | -0.052* | 0.099** | -0.032 | -0.034 | 0.041 | -0.031 |
|  | Nighttime crying | -0.006 | -0.029 | 0.015 | -0.011 | -0.049* | -0.069** | 0.026 | -0.057* | -0.116** |
|  | Allergy | 0.000 | 0.021 | 0.023 | -0.011 | -0.028 | -0.051* | -0.010 | -0.043 | -0.056* |

*P＜0.05, **P＜0.01. P-value＜0.05 significant.
